# Efficacy and safety of dexmedetomidine for prevention of withdrawal syndrome in the pediatric intensive care unit: protocol for an adaptive, multicenter, randomized, double-blind, placebo-controlled, non-profit clinical trial

**DOI:** 10.1186/s13063-019-3793-6

**Published:** 2019-12-11

**Authors:** Maria Cristina Mondardini, Francesca Sperotto, Marco Daverio, Fabio Caramelli, Dario Gregori, Maria Francesca Caligiuri, Francesca Vitale, Maria Teresa Cecini, Marco Piastra, Aldo Mancino, Andrea Pettenazzo, Giorgio Conti, Angela Amigoni

**Affiliations:** 1 Department of Woman, Child and Urological Diseases, Pediatric Intensive Care Unit, University-Hospital S.Orsola-Malpighi Policlinic, Via Albertoni 15, 40138 Bologna, Italy; 2 Department of Woman’s and Child’s Health, Pediatric Intensive Care Unit, University-Hospital of Padua, Via Giustiniani 2, 35128 Padua, Italy; 3 Unit of Biostatistics, Epidemiology and Public Health, Department of Cardiac, Thoracic, Vascular Sciences and Public Health, University-Hospital of Padua, Via Loredan18, 35131 Padua, Italy; 4 Department of Anesthesia and Intensive Care, Pediatric Intensive Care Unit and Pediatric Trauma Center, Catholic University of Rome, A Gemelli Policlinic, Largo Agostino Gemelli 8, 00168 Rome, Italy

**Keywords:** Dexmedetomidine, Analgesia, Sedation, Withdrawal syndrome, Abstinence syndrome, Pediatric intensive care unit

## Abstract

**Background:**

Prolonged treatment with analgesic and sedative drugs in the pediatric intensive care unit (PICU) may lead to undesirable effects such as dependence and tolerance. Moreover, during analgosedation weaning, patients may develop clinical signs of withdrawal, known as withdrawal syndrome (WS). Some studies indicate that dexmedetomidine, a selective α2-adrenoceptor agonist, may be useful to prevent WS, but no clear evidence supports these data. The aims of the present study are to evaluate the efficacy of dexmedetomidine in reducing the occurrence of WS during analgosedation weaning, and to clearly assess its safety.

**Methods:**

We will perform an adaptive, multicenter, randomized, double-blind, placebo-controlled trial. Patients aged < 18 years receiving continuous intravenous analgosedation treatment for at least 5 days and presenting with clinical conditions that allow analgosedation weaning will be randomly assigned to treatment A (dexmedetomidine) or treatment B (placebo). The treatment will be started 24 h before the analgosedation weaning at 0.4 μg/kg/h, increased by 0.2 μg/kg/h per hour up to 0.8 μg/kg/h (neonate: 0.2 μg/kg/h, increased by 0.1 μg/kg/h per hour up to 0.4 μg/kg/h) and continued throughout the whole weaning time. The primary endpoint is the efficacy of the treatment, defined by the reduction in the WS rate among patients treated with dexmedetomidine compared with patients treated with placebo. Safety will be assessed by collecting any potentially related adverse event. The sample size assuring a power of 90% is 77 patients for each group (total *N* = 154 patients). The study was approved by the Ethics Committee of the University-Hospital S.Orsola-Malpighi of Bologna on 22 March 2017.

**Discussion:**

The present trial will allow us to clearly assess the efficacy of dexmedetomidine in reducing the occurrence of WS during weaning from analgosedation drugs. In addition, the study will provide a unique insight into the safety profile of dexmedetomidine.

**Trial registration:**

ClinicalTrials.gov, NCT03645603. Registered on 24 August 2018.

EudraCT, 2015–002114-80. Retrospectively registered on 2 January 2019.

## Background

Analgesia and sedation are essential treatments required by the majority of children admitted to the pediatric intensive care unit (PICU). In addition to their favorable effects, a prolonged exposure to analgosedation drugs may lead to undesirable effects, such as dependence, tolerance and withdrawal syndrome (WS) [1–3]. WS is a clinical syndrome occurring after discontinuation or during weaning from opioids or benzodiazepines, with an incidence ranging from 17 to 57%, up to 64.6% among patients undergoing 5 days or more of treatment [4]. The syndrome is characterized by central nervous system excitement, gastrointestinal disturbance and sympathetic system activation. Typical symptoms for opioid drugs are tremors, agitation, sleeplessness, inconsolable crying, sweating, yawning, sneezing and diarrhea or vomiting [1–3]. The presence of WS causes intense suffering and increases morbidities and the length of PICU stay [3]. For this reason, several studies in the last decades have been designed to identify WS risk factors and an intense effort has been made to find a possible prevention strategy to avoid the onset of WS [5]. Despite this, no clear strategy has so far been identified and the prevention of WS still remains a challenge for pediatric intensivists.

Over the last decades, some case series have indicated that dexmedetomidine, a selective α2-adrenoceptor agonist, may be useful for the prevention or treatment of WS [6–11]. Finkel et al. [8] first described the successful use of dexmedetomidine to allow rapid weaning from conventional analgosedation in two children after cardiac transplantation. Baddigam et al. [9], in the same year, reported the successful use of dexmedetomidine for the treatment of WS in three patients after cardiac surgery. Also, Tobias [10, 11] described the use of intravenous or subcutaneous dexmedetomidine for the prevention of WS in seven children in the PICU, with satisfying results. However, up to now, no high-level evidence studies have supported the role of dexmedetomidine in WS prevention.

We conceived a prospective randomized controlled trial with the aim to evaluate the efficacy of dexmedetomidine in reducing the occurrence of WS during weaning from conventional analgesic and sedative drugs.

## Methods

### Design

**Fig. 1:**
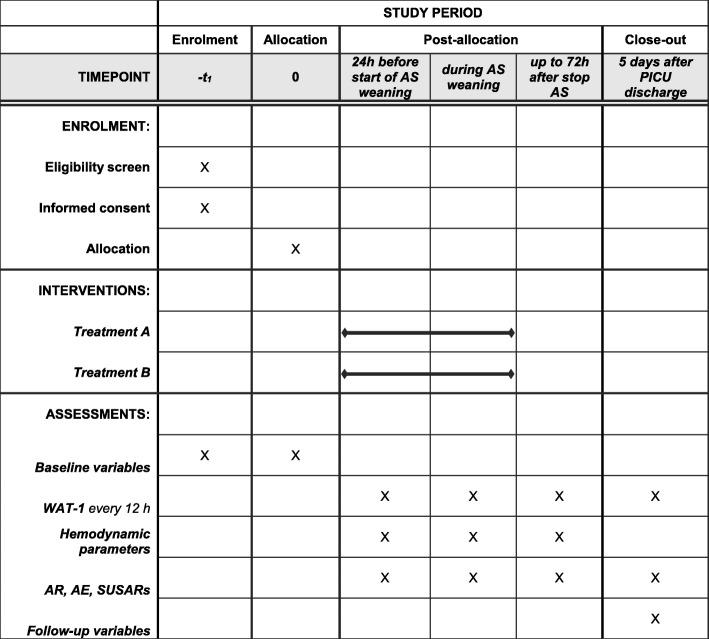
Standard Protocol Items: Recommendations for Interventional Trials (SPIRIT) figure: schedule of enrolment, interventions and assessments of the trial. AE adverse event, AR adverse reactions, AS analgosedation, PICU pediatric intensive care unit, SUSAR serious adverse events and suspected unexpected serious adverse reaction, treatment A receiving dexmedetomidine, treatment B receiving placebo, WAT-1 Withdrawal Assessment Tool version 1

The present study is an adaptive, multicenter, randomized, double-blind, placebo-controlled, non-profit, superiority clinical trial with two parallel groups. The study will be conducted in adherence to the principles of the World Medical Association’s Declaration of Helsinki. An internal Data and Safety Monitoring Board (DSMB) has been nominated to monitor data and safety, and will decide on the continuation, modification or termination of the trial, following the most recent National Institute of Health (NIH) guidelines. The DSMB has no financial or non-financial conflict of interests, as requested by the NIH regulation. The Study Protocol Final Version 2.0 (18 September 2016) was approved by the Ethics Committee of the Coordinating Center (University-Hospital S.Orsola-Malpighi of Bologna) on 22 March 2017. All centers received the approval from the local ethical committee. The study was authorized by the Italian Medicines Agency (AIFA, ID TIP-15-01) and registered in the National Monitoring Center for Clinical Trial (OsSC) and successively in the Eudra CT Register (Identification Number 2015–002114-80). In addition, the study was prospectively registered on the ClinicalTrial.gov registry (registration date 24 August 2018) with Identification Number NCT03645603. The protocol has been designed following the SPIRIT international guidelines: Fig. 1 shows the SPIRIT schedule of enrolment, interventions and assessments, and a populated SPIRIT Checklist is presented in Additional file 1.

### Setting

The study will involve three PICUs belonging to three tertiary-care pediatric academic centers (University-Hospital S.Orsola-Malpighi Policlinic, Bologna, Italy; Catholic University of Rome A. Gemelli Policlinic, Rome, Italy; and University-Hospital of Padua, Padua, Italy).

### Study population

**Fig. 2:**
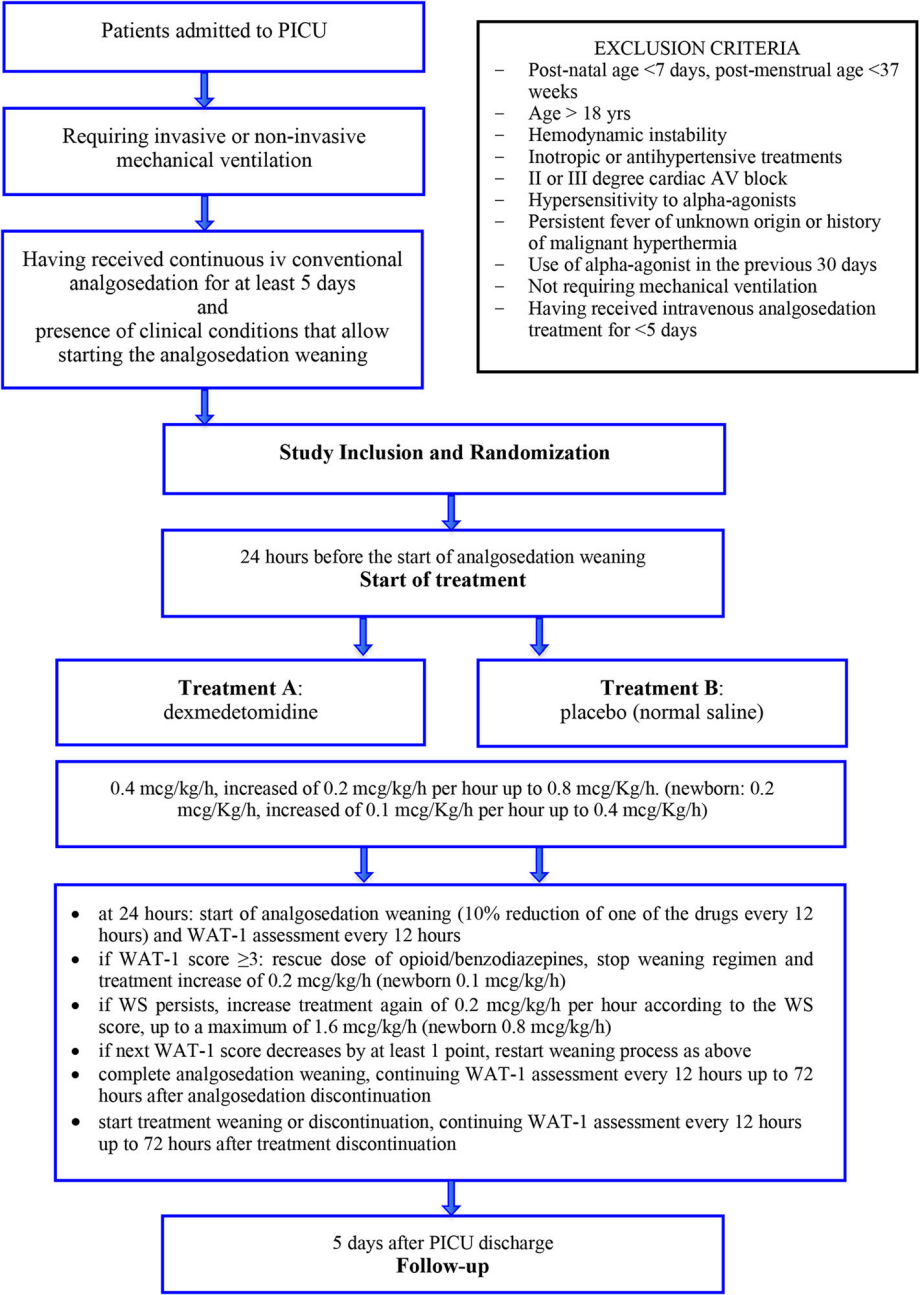
Study protocol flow chart. AV atrio-ventricular, iv intravenous, PICU pediatric intensive care unit, WAT-1 Withdrawal Assessment Tool version 1, WS withdrawal syndrome

The study population will involve patients admitted to the PICU who meet the following criteria (Fig. 2):

- Inclusion criteria: age from 0 to 18 years; postnatal age ≥ 7 days and postmenstrual age (gestational age at birth (weeks) plus weeks since birth) ≥ 37 weeks; having received continuous intravenous analgosedation with opioids and/or benzodiazepines for at least 5 days; having required invasive or non-invasive mechanical ventilation; presence of clinical conditions that allow the treating physician to start the analgosedation weaning, including absence of signs and symptoms of WS; and parents’ written consent obtained.
- Exclusion criteria*:* presence of hemodynamic instability according to the treating physician’s judgment; receiving inotropic or antihypertensive treatments (β-blockers, calcium antagonists, ACE inhibitors, digoxin, nicardipine, nitroglycerin); presence of second or third-degree cardiac atrio-ventricular (AV) block; known or suspected hypersensitivity to α-agonists; presence of persistent unknown-origin fever or history of malignant hyperthermia; and use of α-agonist (clonidine or dexmedetomidine) in the 30 days preceding the study enrolment.

### Definitions

Withdrawal syndrome (WS) is defined as an iatrogenic clinical syndrome that manifests when the administration of a sedative or analgesic agent is abruptly discontinued or too rapidly weaned in a patient who is physically tolerant [2].

The Withdrawal Assessment Tool version 1 (WAT-1) is a validated assessment tool for the monitoring of withdrawal symptoms in pediatric patients. This twice-daily assessment consists of 11 items, determined by the following components: a review of the patient’s record for the past 12 h, a direct observation of the patient for 2 min, a patient assessment using a progressive stimulus and an assessment of post-stimulus recovery [12]. The score ranges from 0 to 12 and a score ≥ 3 indicates the presence of signs or symptoms of WS. The severity of WS is higher as the score increases, as defined by the WAT-1 official definition [12].

### Recruitment and consent

Comprehensive information will be provided by each Center Principal Investigator to the parents of children potentially involved. A detailed information sheet has been designed to support the oral communication. A written informed consent will be obtained from both parents for each involved child. Even when appropriate for age, a child’s consent will be not needed because of the sedation status. A guarantee of optimal children’s care will be assured independently of the study involvement. If present, a consent refusal will be recorded.

### Randomization

Each patient will be randomly assigned to one of the two treatment groups: treatment A group (receiving dexmedetomidine) or treatment B group (receiving placebo). An identification code will be individually assigned to each patient. The Investigational Drug Service of the Coordinating Center generated a block randomization scheme on 11 June 2017 using the website Randomization.com (available online http://randomization.com). This confidential document will be available only to the non-blinded staff, who will carry out the preparation of the treatments. Thus, the allocation sequence and the treatment administration will be unknown to the blinded researchers, including the study Principal Investigator. During the study, two sealed copies of the randomization list that clearly show the treatment attributed to the patient will be available for emergencies. A sealed list will be kept in the archive of the Investigational Drug Service of the Coordinating Center and the others in the archive of each Principal Investigator. If an opening is needed, the Investigator will be asked to report the reason and the date/time of the opening, and to immediately notify the Project Principal Investigator.

### Interventions

Twenty-four hours before the start of analgosedation weaning, an intravenous infusion of dexmedetomidine or placebo (i.e. normal saline) will be started according to the following schedule (Fig. 2). The starting dose will be 0.4 μg/kg/h. No loading dose will be administered. If the infusion is well tolerated (i.e. without the occurrence of adverse effects), the dose will be increased by 0.2 μg/kg/h per hour up to 0.8 μg/kg/h. Given the pharmacological peculiarities of the neonatal period [13], newborns will receive a starting dose of 0.2 μg/kg/h, which will be increased by 0.1 μg/kg/h up to 0.4 μg/kg/h. At 24 h of dexmedetomidine infusion, the analgosedation weaning process will be started, consisting of a 10% reduction of one of the drugs every 12 h. If requested, a switch from opioid and/or benzodiazepine to an equipotent drug of the same pharmacological class but with a longer half-life will be allowed (including enteral methadone, morphine, lorazepam). The switch should aim to facilitate the patient’s management. In the same way as i.v. drugs, enteral drugs will be weaned with a 10% reduction every 12 h.

The WAT-1 scale will be administered every 12 h of treatment infusion. If WS is diagnosed, the clinician will administer a rescue dose of the used opioid and/or benzodiazepine, repeatable until resolution of the crisis, and will increase the dexmedetomidine/placebo dose by 0.2 μg/kg/h (0.1 μg/kg/h in neonates). If the following WAT-1 score shows a decrease by at least 1 point compared with the previous one, the weaning program will be restarted (by 10% of reduction) and the current dexmedetomidine/placebo dosage will be maintained. If the WS symptoms persist, dexmedetomidine/placebo will be increased by 0.2 μg/kg/h (0.1 μg/kg/h in neonates) according to the WS score, up to a maximum of 1.6 μg/kg/h (0.8 μg/kg/h in neonates).

Once the analgosedation weaning is completed, dexmedetomidine will be weaned or discontinued. A gradual reduction of the dexmedetomidine dose is strongly recommended to prevent the risk of dexmedetomidine withdrawal [14, 15], but it is not mandatory. Since the analysis of dexmedetomidine weaning is not a specific aim of the present study, no specific protocol will be recommended. The time and modality of dexmedetomidine weaning will be recorded.

A follow-up visit will be performed at 5 days after PICU discharge, with the aim to collect the following data: actual duration of the analgosedation weaning when longer than 5 days; values of WAT-1 scores collected every 12 h up to 72 h after the analgosedation discontinuation; length of dexmedetomidine weaning (hours); and occurrence of signs and symptoms of dexmedetomidine withdrawal.

### Outcome measures

#### Primary outcome measure

The primary outcome measure of our study is the efficacy of the treatment in the prevention of WS, that is, the reduction of the WS rate in the DEX arm compared with the placebo arm.

#### Secondary outcome measures

##### Treatment safety

The safety of the treatment will be assessed: with strict monitoring of hemodynamic parameters (heart rate, systolic and diastolic blood pressure), which are considered altered if their values differ more than 20% compared with the patient’s baseline values; and collecting every adverse reaction (AR), adverse event (AE), serious AE and suspected unexpected serious adverse reaction (SUSAR). Every AR, AE or SUSAR potentially related to dexmedetomidine will be summarized separately.

##### Secondary outcome measures related to efficacy

Secondary outcome measures evaluated to confirm the efficacy of the treatment will be: trend of the WAT-1 score; number of rescue doses required for WS symptoms; number of temporary discontinuations of the analgosedation weaning due to the presence of WS; duration of analgosedation weaning (days); length of mechanical ventilation (days); and PICU length of stay (days).

### Data collection and management

The blinded investigators will collect data by means of a standardized paper case report form (CRF). Paper CRFs will be stored in accordance with national regulations. Paper CRFs will have an identifiable patient code in order to allow a clinical follow-up and data monitoring by national coordinators or regulatory committees. Investigators will transcribe patient’s data into an electronic CRF using the identification code. No patients’ identifiable data will be directly accessible from the electronic CRF. Data recorded on each CRF will be entered into a dedicated database, checked and subsequently processed.

### Sample size

The sample size has been calculated with respect to our primary outcome measure, that is, the reduction of the WS rate. A recent multicenter national study reported a WS incidence of 64.6% among PICU patients receiving more than 5 days of analgosedation with opioids and/or benzodiazepines [4]. Given the small level of available evidence, to have a clearer picture of the potential reduction of WS, we decided to apply a classical prior elicitation process in four separate stages [16, 17]: selecting the experts and identifying the aspects of the problem to elicit; proceeding with the elicitation process, that is, interaction with the experts; fitting the probability distributions to the expert’s summaries; and including the information from the elicitation process in the evaluation of the sample size requirements.

#### Prior elicitation process

Twenty-eight pediatric intensivists and nurses expert in the field of analgesia and sedation were asked via an email survey about: whether they believe that DEX has some efficacy in reducing WS prevalence; and if yes, what is the expected reduction in WS prevalence via DEX compared to standard care (placebo in the terms of this trial). All of the experts replied to the survey, and 25 (92%) of them declared to expect DEX to have an effect in WS prevalence: 23 of them declared that DEX is able to reduce the WS prevalence, two of them declared that DEX is not able to prevent WS and the last three were not able to provide a certain answer. Among those who replied affirmatively (*n* = 23), 18 provided an estimated percentage of WS reduction in the DEX arm vs. placebo. The median expected reduction in WS prevalence was 47.5% (first quartile 33.75% and third quartile 51.25%).

#### Adaptive design

We assumed that the WS prevalence, according to the literature [4], amounts to about π_Placebo_ = 64.6% and this value was used for the sample size calculation. According to the prior elicitation process, we conservatively adopted the most pessimistic evaluation provided by the experts, assuming a reduction in WS prevalence of 33.75 points in the DEX group compared to placebo. The sample size has been computed using an adaptive approach based on a two-stage group sequential design with an interim sample size reassessment in order to compensate for discrepancies between expected and observed incidences of the primary endpoint at the first stage [18].

The following assumptions were considered:

- Two-sided superiority *Z* test without continuity correction for rejecting the null hypothesis H_0_: π_Placebo_ – π_DEX_ = 0
- α = 0.05
- Power 0.90
- Efficacy bounds derived using an O’Brien–Fleming boundary
- No futility bounds
- An incidence rate of π_Placebo_ = 0.646 in the placebo arm and π_DEX_ = 0.375 in the DEX arm (corresponding to a 33.75% reduction out of the 64.6%)
- An allocation rate of 1:1
  Based on these assumptions, this yields 138 patients overall. To account for a possible *R* = 5% dropout rate, the sample size *n* has been increased [19] to *N* = 138 / (1 – *R*^2^) = 154 total patients. Thus, the trial has been designed as 20 + 20 = 40 patients at the first stage and 154 overall (Additional file 2: Table S1).

##### Sample size reassessment

The sample size reassessment will be performed by the DSMB statistician, who is not involved in the conduct of the clinical trial, once the absence of financial and non-financial conflicts of interests have been confirmed. A promising zone design has been considered. The following steps have been provided at the first interim analysis step:

1. If the computed *z*-test statistic crosses the O’Brien–Fleming Bounds, then the study will be terminated early for efficacy (i.e. |*z*| > 3.92).
2. If the computed *z*-test statistic does not cross the O’Brien–Fleming Bounds, then different solutions are possible:
  - If interim conditional power (CP) is in the promising zone, say comprised between 0.3 and 0.9, then a sample size reassessment will be performed.
  - Otherwise, the study will continue until the second stage.
  - If the CP is critically lower than 0.3, then the internal DSMB will be consulted to discuss a termination of the trial for futility reasons.

A simulation has been performed to evaluate the extent of a sample size reassessment assuming that the null hypothesis will not be rejected at the first interim analysis, and assuming that the interim conditional power is in the promising zone.

To this purpose, reductions in the expected difference in the interim primary endpoint are investigated. A range of interim event rates, say ${p}_{Placebo}^{interim}$ from 0.5 to 0.75 following a pace of 0.01, has been hypothesized in the placebo arm. The interim event rate in the DEX arm is supposed equal to ${p}_{DEX}^{interim}=$ $0.6625\ast {p}_{Placebo}^{interim}$, where 0.6625 corresponds to the most pessimistic reduction hypothesized above.

For each scenario, the number of patients to add to the sample size at the second stage (*n*_*add*_), given the interim results, is computed to achieve a conditional statistical power of at least 0.8 (Additional file 2: Table S2).

Sample size estimation has been performed using gsDesign [20] and the R system [21].

### Statistical analysis

The primary endpoint will be analyzed for the intent-to-treat (ITT) population in terms of the WS rate (placebo vs. dexmedetomidine). If the interim conditional power is not in the promising zone, no modifications to the design will be performed.

The final efficacy of the treatment will be evaluated using *Z*-test statistics; after having reached the sample size foreseen for each of the stages, an evaluation using a 95% repeated confidence interval will be performed.

Demographics (age, sex and race) and other baseline characteristics will be summarized using descriptive statistics. As for secondary outcome measures, no formal testing procedure will be adopted, to avoid the inflation of type I errors. Differences in distributions will be evaluated on a clinical reasoning basis.

An analytical detailed list of patients who will discontinue the study for ARs, AEs or SUSARs will be collected.

All analyses will be performed using the R system [21].

### Trial status

The present trial (Study Protocol Final Version 2.0, approved on 18 September 2016) is currently ongoing. All centers are actively recruiting patients. From the beginning of the enrolment (30 August 2018) to date (22 July 2019), 34 of 160 patients have been recruited. The period for the whole population enrolment has been estimated as 3 years (estimated end-of-enrolment date: August 2021).

## Discussion

To the best of our knowledge, this is the first randomized prospective controlled trial addressing the important issue of WS prevention using dexmedetomidine, a highly selective α2-agonist with unique pharmacological properties.

WS represents one of the most important causes of morbidity in patients receiving prolonged sedation in the PICU [3], and several studies have so far been conducted to identify the main risk factors and the most successful prevention strategy. The cumulative dose of the analgosedation drug and the duration of treatment have been described as main factors associated with the onset of WS, as well as the rapid dosage reduction and the abrupt discontinuation of the treatment [5]. Therefore, WS prevention strategies have addressed both restriction of drug exposure and tapering of the infusion. A strategy of drug switching has been also proposed, replacing the used drug with another equipotent drug with a longer half-life. Although no drug seems more effective than others, methadone is the most commonly prescribed [22]. However, the efficacy of these strategies in preventing WS is still unclear.

In the past decades, dexmedetomidine has been suggested as a useful strategy to prevent WS [6–11]. Binding the α2-receptor, dexmedetomidine is able to block the release of noradrenaline in the locus coeruleus, mediating a sedative and anxiolytic effect [1], and to block substance-P release in the dorsal horns of the spinal cord, mediating a mild analgesic effect [23–26]. The sympathetic inhibition is also responsible for the most common AEs, such as hypotension and bradycardia, usually easily reversible with dose reduction [27, 28]. The concept that dexmedetomidine could have potentiality in WS management originated from the knowledge of other α2-receptor agonists (i.e. clonidine and lofexidine) used in the adult population [6, 29]. In fact, the ability of α2-agonists to interact with the sympathetic system represents the pharmacological rationale for their use as adjuvant drugs for the management of WS, which is characterized by sympathetic activation [29]. The interaction between dexmedetomidine and opioids was first described in murine models treated with morphine prolonged infusions and induced to present WS [30]. The authors described that dexmedetomidine and opioids seem to have a reciprocal adjuvant effect to induce both analgesia and hypnosis. During opioid WS, dexmedetomidine maintains its hypnotic effect even if its analgesic effect and the morphine-reciprocal effect decrease [30]. Other than these preclinical results, studies on dexmedetomidine for prevention of WS have been limited to case series [6–11], and its clinical use in pediatrics has been limited by its off-label status.

The present trial will aim to systematically analyze the efficacy of dexmedetomidine for prevention of WS in pediatric patients receiving a prolonged analgosedation treatment. Its multicenter, randomized controlled design will allow us to clearly assess this research question with a high level of evidence. The systematic evaluation of WS by means of a standardized score validated for pediatric age, that is, the Withdrawal Assessment Tool version 1 (WAT-1) [12], will ensure precision in the WS registration and increase the validity of the study, as well as its reproducibility. Moreover, the feasibility of the present trial will be guaranteed by the fact that dexmedetomidine is easily available in most of the tertiary-care pediatric centers in Europe, as well as the other resources requested for the implementation of the study. In the case that the efficacy of dexmedetomidine is proven, the dexmedetomidine arm of the present trial could be translated into a successful WS-prevention protocol, offering a real opportunity to adequately approach one of the biggest challenges of prolonged sedation in the PICU. In addition, the present study will measure whether the use of dexmedetomidine could reduce the duration of conventional analgosedation, mechanical ventilation and PICU stay with a possible impact on PICU-resources management. Finally, the trial will systematically evaluate any kind of dexmedetomidine-related adverse events, particularly hemodynamic ones, providing a unique insight into its safety profile.

Our study is also subject to limitations. In fact, patient selection and protocols for conventional analgesia and sedation and for WS treatment were not standardized, and are therefore subject to practice variability. The randomization is not balanced among centers, precluding the control of a possible center effect. The most severe patients could be difficult to enroll due to the complexity of maintaining commitment to the protocol. Finally, the follow-up could be subject to missing data if the discharge includes a transfer to another center or institution. Despite these limits, we believed that this trial could represent an important step in the definition of a new strategy for the prevention of WS in critically ill children.

## Supplementary information

**Additional file 1.** This additional file provides the populated SPIRIT Checklist for the Study Protocol, as requested by the SPIRIT guidelines.

**Additional file 2: **This additional file provides supplemental material regarding the statistical methodology. **Table S1.** Summary information for the group sequential O'Brien–Fleming design. **Table S2.** Sample size reassessment calculation under various hypothetical scenarios.

## Data Availability

The Principal Investigator (MCM) and the authors will have full access to the final dataset data during the analysis. The datasets used and analyzed during the current study are available from the corresponding author on reasonable request.

## Abbreviations

AE: Adverse event
AR: Adverse reaction
AV: Atrio-ventricular
CRF: Case report form
PICU: Pediatric intensive care unit
SUSAR: Suspected unexpected serious adverse reaction
WAT-1: Withdrawal Assessment Tool version 1
WS: Withdrawal syndrome
