# Supplementary material for: Efficacy and safety of dexmedetomidine for prevention of withdrawal syndrome in the pediatric intensive care unit: protocol for an adaptive, multicenter, randomized, double-blind, placebo-controlled, non-profit clinical trial
Source: Trials. 2019 Dec 11;20:710. doi: 10.1186/s13063-019-3793-6 (PMC6907190; doi:10.1186/s13063-019-3793-6)
Supplement: Supplementary file 2 — Additional file 2: This additional file provides supplemental material regarding the statistical methodology. Table S1. Summary information for the group sequential O'Brien–Fleming design. Table S2. Sample size reassessment calculation under various hypothetical scenarios. [file 13063_2019_3793_MOESM2_ESM.docx]

**Additional file 2**

**Table S1.** Summary information for the Group Sequential O'Brien-Fleming design

|  | Information Rate | Bounds to reject H_0_ | Significance level  (one-sided) | α spent | Power achieved | Placebo | DEX | N |
| --- | --- | --- | --- | --- | --- | --- | --- | --- |
| **Stage 1 (Interim)** | 0.25 | 3.92 | 0.00001 | 0.00001 | 0.025 | 20 | 20 | 40 |
| **Stage 2 (Final)** | 1.0 | 1.96 | 0.025 | 0.025 | 0.9 | 57 | 57 | 154 |

**Table S2.** Sample size reassessment calculation under various hypothetical scenarios.

| $p_{Placebo}^{interim}$ | $p_{DEX}^{interimto}$ | *n* to add | Conditional Power | Overall *N* |
| --- | --- | --- | --- | --- |
| 0.75 | 0.53 | 0 | 0.93 | 154 |
| 0.74 | 0.52 | 0 | 0.92 | 154 |
| 0.73 | 0.51 | 0 | 0.91 | 154 |
| 0.72 | 0.50 | 0 | 0.90 | 154 |
| 0.71 | 0.50 | 0 | 0.89 | 154 |
| 0.70 | 0.49 | 0 | 0.88 | 154 |
| 0.69 | 0.48 | 0 | 0.87 | 154 |
| 0.68 | 0.48 | 0 | 0.85 | 154 |
| 0.67 | 0.47 | 0 | 0.84 | 154 |
| 0.66 | 0.46 | 0 | 0.83 | 154 |
| 0.65 | 0.46 | 0 | 0.81 | 154 |
| 0.64 | 0.45 | 2 | 0.80 | 157 |
| 0.63 | 0.44 | 6 | 0.80 | 161 |
| 0.62 | 0.43 | 12 | 0.80 | 167 |
| 0.61 | 0.43 | 18 | 0.80 | 173 |
| 0.60 | 0.42 | 24 | 0.80 | 179 |
| 0.59 | 0.41 | 30 | 0.80 | 185 |
| 0.58 | 0.41 | 36 | 0.80 | 191 |
| 0.57 | 0.40 | 42 | 0.80 | 197 |
| 0.56 | 0.39 | 50 | 0.80 | 205 |
| 0.55 | 0.39 | 56 | 0.80 | 211 |
